# Vagus Nerve Stimulation Alleviates Hepatic Ischemia and Reperfusion Injury by Regulating Glutathione Production and Transformation

**DOI:** 10.1155/2020/1079129

**Published:** 2020-01-21

**Authors:** Haoyang Xia, Zhongzhong Liu, Wenjin Liang, Xianpeng Zeng, Yi Yang, Pu Chen, Zibiao Zhong, Qifa Ye

**Affiliations:** ^1^Zhongnan Hospital of Wuhan University, Institute of Hepatobiliary Diseases of Wuhan University, Transplant Center of Wuhan University, Hubei Key Laboratory of Medical Technology on Transplantation, Engineering Research Center of Natural Polymer-Based Medical Materials in Hubei Province, Wuhan 430071, China; ^2^College of Health Science, Wuhan Sports University, Wuhan 430079, China; ^3^Department of Biomedical Engineering, School of Basic Medical Sciences, Wuhan University, Wuhan, Hubei 430071, China; ^4^Transplantation Medicine Engineering and Technology Research Center, National Health Commission, The 3rd Xiangya Hospital of Central South University, Changsha 410013, China

## Abstract

Inflammation and oxidative stress are pivotal mechanisms for the pathogenesis of ischemia and reperfusion injury (IRI). Vagus nerve stimulation (VNS) may participate in maintaining oxidative homeostasis and response to external stimulus or injury. We investigated whether the *in vivo* VNS can protect the liver from IRI. In this study, hepatic IRI were induced by ligating the vessels supplying the left and middle lobes of the liver, which underwent 1 h occlusion followed with 24 h reperfusion. VNS was initiated 15 min after ischemia and continued 30 min. Hepatic function, histology, and apoptosis rates were evaluated after 24 h reperfusion. Compared with the IRI group, VNS significantly improved hepatic function. The protective effect was accompanied by a reduction in histological damage in the ischemic area, and the apoptosis rate of hepatocytes has considerable reduction. To find the underlying mechanism, proteomic analysis was performed and differential expression of glutathione synthetase (GSS) and glutathione S-transferase (GST) was observed. Subsequently, test results indicated that VNS upregulated the expression of mRNA and protein of GSS and GST. Meanwhile, VNS increased the plasma levels of glutathione and glutathione peroxidases. We found that VNS alleviated hepatic IRI by upregulating the antioxidant glutathione via the GSS/glutathione/GST signaling pathway.

## 1. Introduction

Liver transplantation (LT) is an effective treatment for patients suffering from several end-stage liver diseases [1], and this therapeutic regimen has seen important improvements, including machine perfusion [2] and the use of immunosuppressant [3]. Allografts, which are procured via donation after cardiac death (DCD), are considered to be a useful additional source that can cover the shortage of LT. However, compared with ideal donors, increasing evidence indicates that DCD livers are highly vulnerable to ischemia and reperfusion injury (IRI) [4, 5], which is an inevitable process in LT [6]. Hepatic IRI may lead to some complications in the perioperative period of transplantation, including poor graft function [7], liver dysfunction [8], and a high risk of retransplantation [9, 10]. Because of its clinical significance, there are several treatments that have been used for the preservation of allografts, in which cold storage and machine perfusion are predominantly performed. However, these two treatments are implemented after the occurrence of injury. Therefore, an improved therapy that can be performed before or during injury is urgently needed. In the progress of IRI, initial organ damage induced by oxygen and nutrient deprivation during the ischemic period and the subsequent and worse injury during reperfusion are caused by tissue inflammation and oxidative stress. Ischemic preconditioning (IPC) may be a useful means to relieve the symptoms of IRI [11, 12]; however, the beneficial effect of IPC is limited by many factors, including the age of the patients and duration of occlusion [13, 14]. Thus, an effective treatment for donor livers with hepatic IRI, or for other patients with such injury, is clearly needed.

As a promising preservation technique, it has been shown that pulsed ultrasound (US) can protect kidneys from IRI [15], perhaps by invoking an anti-inflammatory response. This is consistent with the regulatory effects of an inflammatory reflex called the cholinergic anti-inflammatory pathway (CAP) [16, 17]. In this reflex, inflammatory regulatory signals are transmitted by the peripheral and central nervous systems. Studies have shown that the inflammatory signaling is produced by nervous stem nuclei of the vagus nerve, and the reflex can be activated by vagus nerve stimulation (VNS) [18]. In addition, VNS has already been approved to treat refractory epilepsy and drug-resistant depression [19, 20]. Besides its therapeutic effect on neuropsychiatric disorders, VNS can play a key role in regulating the CAP reflex to treat inflammatory disorders such as rheumatoid arthritis [21] and inflammatory bowel disease [22].

VNS has also been tested on animal models as a treatment for multiple diseases [23, 24], including IRI [25, 26]. In mitigating IRI using VNS, the cerebrum and myocardium have been intensely studied [27–29], but the effect of VNS on IRI in LT has not been verified. Considering the therapeutic efficacy of VNS on IRI and other illnesses, we hypothesized that VNS can prevent liver IRI. In this study, we used a continuous constant stimulus system and investigated whether vagal stimulation can attenuate IRI in rat livers and revealed the underlying mechanism involved.

## 2. Materials and Methods

### 2.1. Animals

Male Sprague-Dawley rats (8-10 weeks, 250-300 g) were used for experiments. Five rats were used in each group. All rats were fed with a unified standard chow, had free access to food and drinking water, and were housed under a standard interior environment (20-25°C, 50%-70% humidity). All animal experiments were carried out in accordance with the Experimental Animal Care and Use Committee of Zhongnan Hospital and the *Guide for the Care and Use of Laboratory Animals*. The experiments were approved by the Animal Ethics Committee of Zhongnan Hospital of Wuhan University.

### 2.2. Study Design

**Figure 1:**
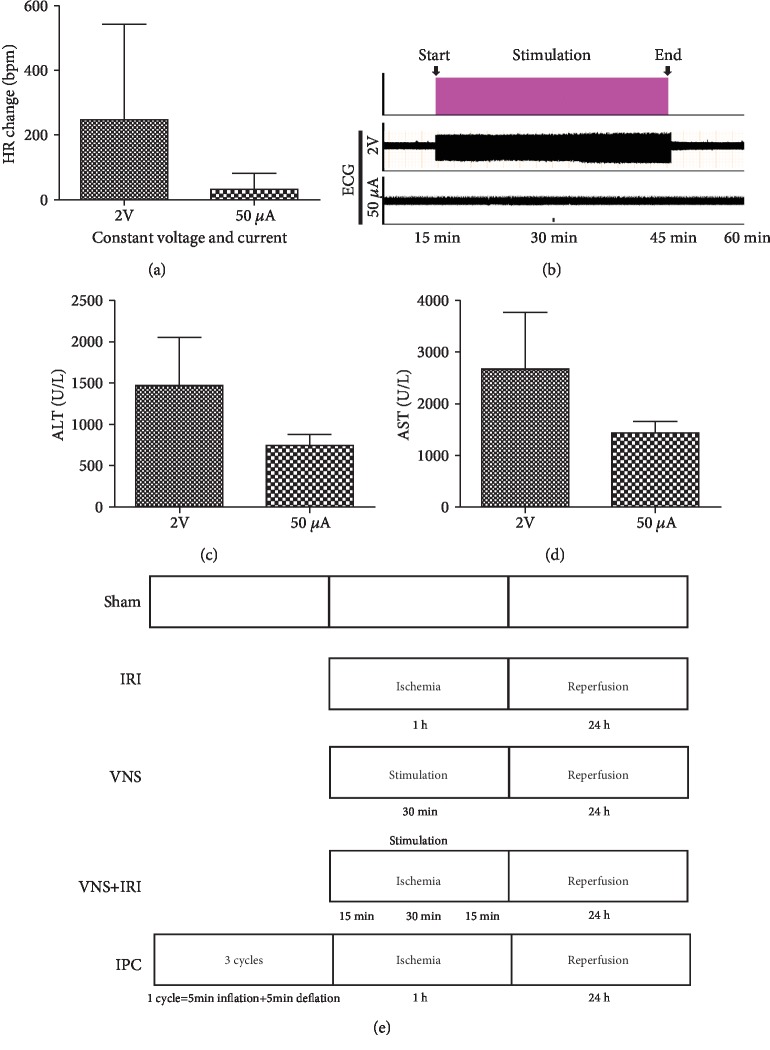
Establishment and optimization of vagus nerve stimulation. Heart rate was recorded as rats underwent vagus nerve stimulation at given parameters (1 ms, 10 Hz), but with different stimulus intensities (2 V, 50 *μ*A). Changes in the heart rate (a) of vagal stimulation compared with ischemia without stimulation. 50 *μ*A of current reduced heart rates more reliably. (b) Electrocardiograph during left vagal stimulation. (c, d) Rats underwent vagal stimulation or sham during the ischemic period, and blood was collected and tested at the end of 24 h of reperfusion. (e) Experimental protocol and established five experimental groups. ^∗∗^*P* < 0.01.

To investigate the protective effect of VNS on hepatic IRI, we compared the extent of injury in pure ischemic livers and ischemic livers which were stimulated by VNS. Meanwhile, the pure VNS group was set to exclude the interference of VNS on physiological conditions. For this purpose, experimental groups were established (Figure 1(e)):

1. *Sham Group*. Healthy livers without ischemia and VNS.
2. *IRI Group*. Livers that were exposed to 60 min *in situ* ischemia and underwent 24 h reperfusion and also without VNS.
3. *VNS Group*. Rats that received VNS alone and without ischemia; these livers were harvested after 24 h reperfusion.
4. *VNS+IRI Group*. Rats that underwent 1 h hepatic ischemia followed with 24 h reperfusion and received VNS which was initiated 15 min after ischemia and continued 30 min.
5. *IPC Group*. Rats that accepted 3 cycles of 5 min inflation/5 min deflation prior to 1 h hepatic ischemia, the obstruction placed on the same vessels of hepatic IRI.

### 2.3. VNS and Miscellaneous Recordings

All rats used to evaluate the effect of VNS on the IRI were given general anesthesia with pentobarbital sodium salt (MilliporeSigma, Burlington, MA, USA). The left vagus nerve was stimulated, because this side of the nerve is frequently selected to be stimulated in animal and human experiments [25]. The left vagus nerve was isolated through a midline cervical incision and contacted with a shielded electrode for stimulation. In all tested rats, the nerve was intact and stimulated by constant electrical stimulation (continuous single stimulation, 50 *μ*A intensity; frequency, 10 Hz; duration, 1 ms) [30], which was applied for 30 min and started at 15 min after hepatic ischemia, using a RM6240 stimulator and stimulus isolation unit (Chengdu Instrument Factory, Chengdu, Sichuan, China). In the sham-operated group, the vagus nerve was exposed but not stimulated.

The stimulation parameters were chosen after evaluating their effects on the heart rate (HR) in preliminary experiments. In anesthetized rats, the HR was recorded from ECG (RM6240, Chengdu Instrument Factory, Chengdu, Sichuan, China) electrodes inserted.

### 2.4. Hepatic IRI

Surgical operations were performed under general anesthesia. A longitudinal abdominal incision was made, and the hepatic ligament was incised. Ischemia of the liver was induced by establishing a temporary occlusion of the pedicle of the left and middle lobes (approximately 70% of the total volume of the liver) using a tiny vascular clamp. After completing the above procedures, the abdomen was temporarily closed during the ischemia period to minimize the effect of dehydration and temperature changes. All groups except the sham group (*n* = 5 in each group) received the same combination of ischemia and reperfusion injury: 60 min of ischemia followed by 24 h of reperfusion. Blood and tissue samples were obtained at the end of the reperfusion period. The liver was lavaged in the same manner in all rats, including the sham group.

### 2.5. Plasma Aminotransferase and Analysis of Tissue Morphology

Plasma was prepared by centrifuging the collected blood samples. Plasma aminotransferase, including alanine aminotransferase (ALT) and aspartate aminotransferase (AST), was analyzed with standard methods at the clinical laboratory of Zhongnan Hospital of Wuhan University.

Liver tissues were fixed with 10% buffered formalin, then embedded in paraffin and cut into 5 *μ*m sections for histologic analysis via hematoxylin and eosin staining (H&E). Section images were acquired by a Leica Microsystems microscope (DM200; Wetzlar, Germany) at ×100 and ×400 magnification. Five horizons of each section were selected randomly to appraise liver damage. According to Suzuki's criteria [31], congestion, vacuolization, and necrosis were scored from 0 to 4.

### 2.6. TUNEL Assays

TUNEL assays were performed with the TUNEL Apoptosis Assay Kit (11684817910, Roche, Shanghai, China). Paraffin-embedded sections were treated with proteinase K (G1205, Servicebio, Wuhan, Hubei, China) for 25 min at 37°C and subsequently incubated with a mixture of fluorescent labeling solution and TdT enzyme at 37°C for 2 h in a humidified environment. Then, samples were washed in PBS and mounted in mounting media that contained DAPI (G1012, Servicebio, Wuhan, Hubei, China). Fluorescent images were captured by an inverted fluorescent microscope (TH4-200; Olympus, Tokyo, Japan) at ×200 magnification. Six horizons of each section were selected randomly for TUNEL assays. The apoptosis rate was calculated according to the following formula: TUNEL‐positive cells (*n*)/total cells (*n*) × %.

### 2.7. Comparative Proteomic Analysis Based on Isobaric Tag for Relative and Absolute Quantitation (iTRAQ) Labeling and Bioinformatics Analysis of Differentially Expressed Proteins

The iTRAQ labeling experiment was carried out as described previously [32]. In the current study, proteins with 95% or greater confidence as determined by ProteinPilot Unused scores were reported, and the corresponding FDR was less than 1%.

Bioinformatics analysis of differentially expressed proteins was performed with Mascot 2.6, Proteome Discoverer 2.1, Blast2GO, InterProScan, Cluster 3.0, Java TreeView, and KEGG Automatic Annotation Server (KAAS) software.

### 2.8. Biochemical Analysis

Reduced (GSH) and oxidized (GSSG) glutathione in the plasma was measured by a spectrophotometric detection method [33–35]. Glutathione peroxidase (GPx) activity was determined indirectly by a coupled reaction with glutathione reductase and the oxidation of NADPH to NADP^+^.

### 2.9. Malondialdehyde (MDA) and Superoxide Dismutase (SOD)

For evaluating the degree of oxidative stress, frozen hepatic tissue was homogenized and determined by using the colorimetric assay. The testing results were measured as nmol/mgprot and U/mgprot, respectively.

### 2.10. Western Blot Analysis

The collected hepatic samples were stored in a low temperature refrigerator at -80°C. Total protein lysates were prepared by hepatic tissue homogenization using RIPA lysis buffer (P0013B, Beyotime, Shanghai, China). Cytoplasmic proteins were prepared, and the concentrations of tissue samples were determined by bicinchoninic acid protein concentration assays (P0010, Beyotime, Shanghai, China). SDS-PAGE was performed, and proteins were transferred to PVDF membranes followed by incubation at 4°C overnight with primary antibodies at the corresponding concentrations: glutathione synthetase (GSS, GR79078-9, 1 : 5000, Abcam, Shanghai, China), glutathione S-transferase (GST, AE006, 1 : 1000, ABclonal, Shanghai, China). Membranes were then washed with Tris-buffered saline-Tween repeatedly and incubated with secondary antibodies at room temperature for 1 h. Immunoreactive bands were visualized using an ECL kit method as previously described [27]. GAPDH (60004-1-lg, 1 : 8000, Wuhan Proteintech Group, Wuhan, Hubei, China) was used as a loading control. Quantification of protein bands was carried out with ImageJ software.

### 2.11. Reverse Transcription-Quantitative Polymerase Chain Reaction

Total RNA was obtained from hepatic tissues with a TRIzol reagent (EX1880, G-CLONE, Beijing, China) according to the manufacturer's protocol, and cDNA for RT-qPCR was synthesized using oligo d(T). qPCR was conducted using a Quantitative SYBR-Green RT-PCR kit (11203ES08, Yeasen Biotech, Co., Ltd., Shanghai, China) and an Applied Biosystems 7500 system (Thermo Fisher Scientific, Inc., Waltham, MA, USA). All reactions were processed in a 20 *μ*L volume in triplicate. Relative expression levels for target genes were normalized to *β*-actin. Specificity was verified by melting curve analysis and agarose gel electrophoresis. Glutathione synthetase, glutathione S-transferase m3, glutathione S-transferase m5, and *β*-actin were obtained from Wuhan TSINGKE Biotechnology (Wuhan, China). Primers were as follows: glutathione synthetase, 5′-ACAACGAGCGAGTTGGGAT-3′ and 5′-TGAGGGGAAGAGCGTGAATG-3′ (reverse); glutathione S-transferase m3, 5′-CACAGAGCGAGAAAGGAGGA-3′ and 5′-CCCAGTAACCCAGAACCATAGA-3′ (reverse); glutathione S-transferase m5, 5′-TGGTTCGGCTCTGCTACA-3′ and 5′-GCACTTGGGCTCAAACATAC-3′ (reverse); IL-1*β*, 5′-GACTTCACCATGGAACCCGT-3′ and 5′-GGAGACTGCCCATTCTCGAC-3′ (reverse); and IL-6, 5′-AGAGACTTCCAGCCAGTTGC-3′ and 5′-AGTCTCCTCTCCGGACTTGT-3′ (reverse). Data was analyzed using the comparative CT (2^-*ΔΔ*CT^) method [36].

### 2.12. Statistical Analysis

The results of several observations are presented as the means ± SD of at least three experiments. Statistical significance was determined using a one-way analysis of variance (ANOVA), and analysis of nonnormal distribution of indicators was performed with the Kruskal-Wallis test. *P* values < 0.05 were considered as statistically significant.

## 3. Results

### 3.1. Animal Model Construction and Stability Evaluation

Stimulating the vagus nerve can induce bradycardia, so therefore, the level of bradycardia in VNS was assessed to evaluate the extent of different stimulus types. Compared with the constant current group, the constant voltage group demonstrated a considerable increasing level of bradycardia, and this variation was very unstable during stimulation, which was reversed in the constant current group (Figure 1(a)). In the meantime, electrocardiographs were recorded during the ischemic period. In contrast to constant voltage stimulation, constant current stimulation did not show a significant change (Figure 1(b)).

Previous studies have shown that VNS can mitigate the IRI response [37]. Hence, after eliminating the effects of stimulation on the circulatory system, stimulation was used to test whether VNS was effective in improving hepatic function following the given protocol (Figure 1(e)). ALT and AST are two common markers that reflect the status of function; we found that significantly lower and more stable levels of ALT and AST were observed in the stimulated group using constant current stimulation (Figures 1(c) and 1(d)). Therefore, we demonstrated that a stimulated animal model, in accordance with the selected parameters (1 ms, 10 Hz, and 50 *μ*A), was properly constructed.

### 3.2. VNS Alleviates Liver Damage and Improves Liver Function

**Figure 2:**
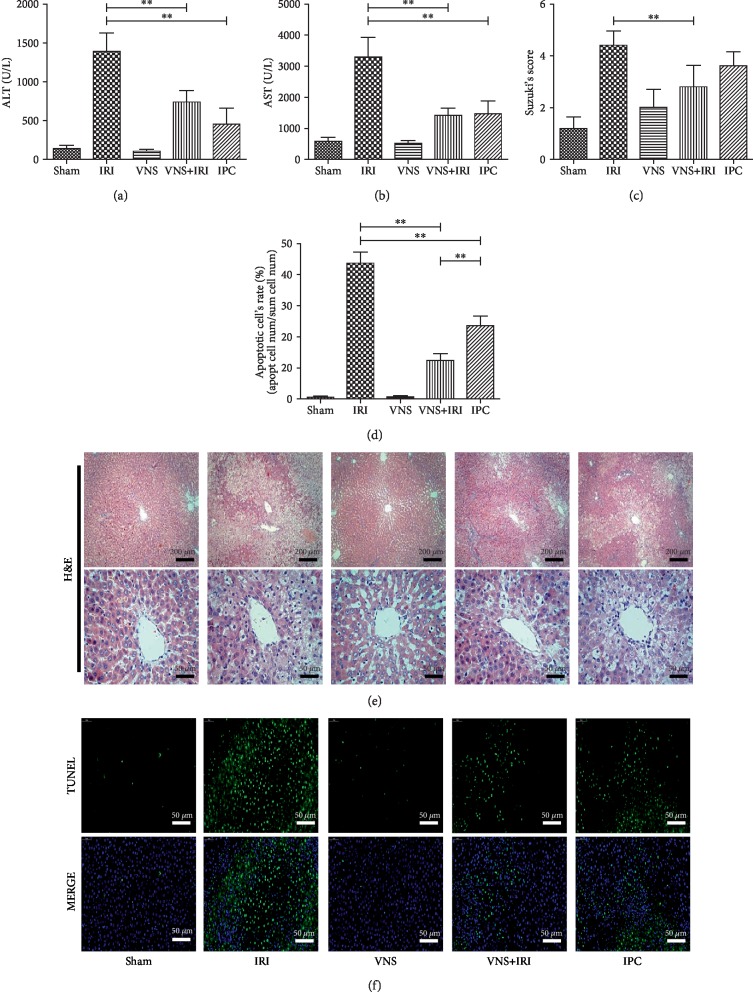
Vagus nerve stimulation during ischemia protects livers from ischemia and reperfusion injury. Rats underwent vagal stimulation or sham surgery, performed on the left vagus nerve, during the ischemia period. Vagal stimulation protected livers and mitigated the ischemia and reperfusion-induced increase of hepatic enzymes, including glutamic-pyruvic transaminase (a) and glutamic oxalacetic transaminase (b). Furthermore, vagal stimulation alleviates damage of hepatic tissues ((c) Suzuki's scores obtained from H&E samples). (d) Vagal stimulation during the ischemic period alleviates apoptosis from IRI. (e) Representative H&E profiles of hepatic samples. (f) Representative TUNEL stain images in liver tissue sections from hepatic IRI in rats. *N* = 5 in each group. ^∗∗^*P* < 0.01. Scale bars: 200 *μ*m and 50 *μ*m.

The level of ALT and AST enzymes in plasma was detected to determine the extent of hepatic IRI. Compared to the sham group, the IRI group had significantly increased levels of ALT and AST enzymes after reperfusion, which were reversed in the VNS+IRI group (Figures 2(a) and 2(b)). The level of ALT was decreased from 1387.8 ± 236.4 U/L in the IRI group to 738.8 ± 143.3 U/L in the VNS+IRI group, and similarly, the level of AST also declined, from 3289 ± 656.67 U/L to 1421.6 ± 226.7 U/L. Meanwhile, it is known that the IPC may be a useful means to relieve the damage of IRI; therefore, the protective effect of vagal stimulation and IPC was compared to further investigate the effects of VNS. Although there was no statistically significant difference in the VNS+IRI and IPC groups, hepatic enzymes showed much the same effect in these two different intervention groups.

Histologic evaluation confirmed that, after reperfusion, hepatic IR-induced injuries in the sham group were not severe, meaning slight congestion and vacuolization were occasionally found. In the IRI group, there was severe congestion in hepatic sinusoids, and most hepatocytes were observed to have vacuolization (Figure 2(e)). Furthermore, hepatocytes of the IRI group had distinctive nucleus changes, including karyolysis and pyknosis, and eosinophilic and granular changes were seen in the cytoplasm. Compared with the other parts of the liver, IR-induced injury of the pericentral region was striking.

In the VNS+IRI group, after being stimulated, hepatic damage was observed to be in significant remission. To analyze the status of injuries of livers in different groups, we used Suzuki's assessment. Although the protective effect could also be achieved by IPC, we found that the VNS+IRI group decreased more damage than the IPC group and was statistically different compared with the IRI group; therefore, according to the damage of hepatic tissue, the VNS+IRI group can reduce the IR-induced damage more than IPC (Figure 2(c)). Previous studies have shown that VNS and CAP can provide protection from inflammation and injuries [38, 39]. We determined the apoptosis rate of livers among groups after 24 h of reperfusion. Compared with the sham group, hepatocyte apoptosis was remarkably higher in the IRI group; however, ischemic livers receiving VNS resulted in a marked decrease in apoptosis. In addition, the VNS+IRI group had a lower rate of apoptosis than the IPC group, and this difference was statistically significant (Figures 2(d) and 2(f)). Therefore, our finding hinted that VNS could protect the livers from IRI; moreover, it is better than IPC in some respects, which is commonly used to alleviate IRI.

### 3.3. iTRAQ Analysis of IRI and VNS+IRI Rats

**Figure 3:**
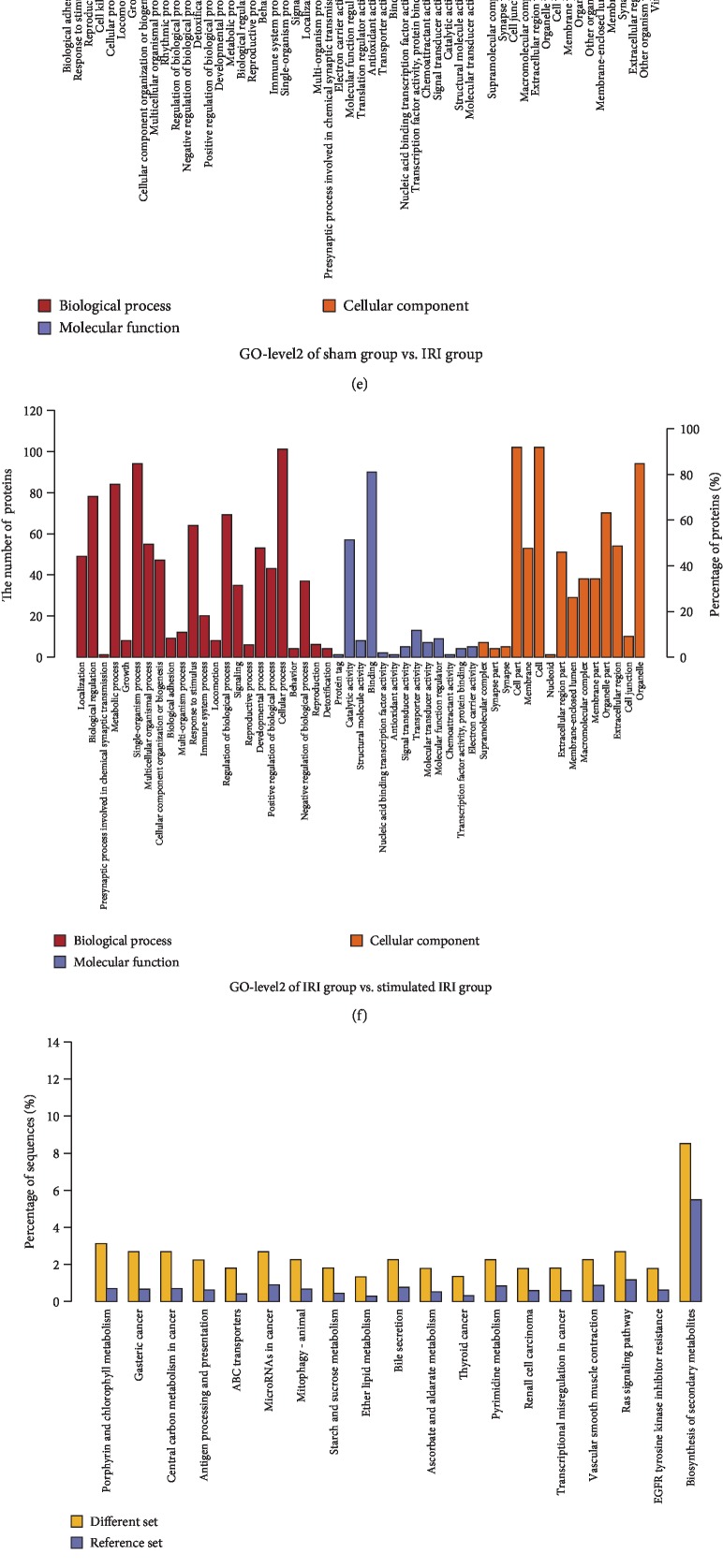
Proteins up- and downregulated in livers by IR and VNS performed during the ischemic period. Rats underwent VNS or sham procedure during ischemia, and then, proteins were isolated from hepatic tissues and proteomic analysis was performed. Altered expressions of IRI and VNS+IRI groups, compared with sham or IRI groups, respectively, were calculated to generate detailed profiles. (a) Volcano plot of the IRI group compared with the sham group. (b) Volcano plot of the VNS+IRI group compared with the IRI group. (c) Cluster analysis of the IRI group compared with the sham group. (d) Cluster analysis of the VNS+IRI group compared with the IRI group. Detailed information of proteins altered by vagal stimulation. Rats underwent vagal stimulation or sham surgery during the ischemic period. Proteins were isolated from hepatic tissues, and isobaric tags for relative and absolute quantification were performed. (e, f) Detailed information of up-/downregulated proteins was analyzed by bioinformatics (sham group vs. IRI group and IRI group vs. VNS+IRI group, respectively) and was performed to generate two GO-level2 maps. (g, h) Possible pathways of the same comparison were also evaluated, generating two Kyoto Encyclopedia of Genes and Genomes enriched maps.

To elucidate the underlying mechanism by which VNS reduces hepatic IRI, we performed quantitative proteomic analysis-based iTRAQ. Similarly, to identify proteins that were either up- or downregulated during vagal stimulation, the relative expressions of proteins were compared between the sham and IRI groups and the IRI and VNS+IRI groups. We then visualized the results. As shown in Figure 3, there were more than 100 proteins that were differentially expressed in the VNS+IRI group compared with the IRI group in the three independent biological replicates (FDR < 1%). We noted possible signaling pathways and merged the proteins that have the same accession and gene name to identify nonredundant proteins. Detailed information about the identified proteins and possible pathways is displayed in Figures 3(e)–3(h).

Afterward, mass spectrometry was performed to evaluate the quality and biological reproducibility. Quantitative and SDS-PAGE results showed that the protein was of good quality, the total amount was sufficient, and the reproducibility between samples was good. In addition, protein mass spectrometry showed normal enzymatic hydrolysis and normal chromatography-mass. To determine proteins that were regulated during vagal stimulation, the fold changes of protein expression were identified. Accordingly, there were dozens of proteins changed significantly in stimulated vagus nerves, suggesting a drastic alteration when VNS was performed.

### 3.4. Confirmation of Previous Findings

Through iTRAQ analysis, as shown in Figures 3(e)–3(h), up- and downregulated proteins were discovered to be involved in several pathways. Then, combined with the identification of possible pathways, there were five regulated proteins that are involved in glutathione metabolism, which typify the characteristics of free radical scavenging associated with glutathione. The GSS included in these proteins is a synthetase, not a rate-limiting enzyme of glutathione, and GSTm3 and GSTm5 were also found, implicating antioxidation of various free radicals by binding of nucleophilic glutathione. Thus, the changed proteins demonstrated by the present proteomic experiment are, a great extent, related to glutathione biosynthesis and metabolism.

### 3.5. VNS Improved GSH, GSSG, and GPx Activities in the Liver, with Reduction of Inflammation Cytokines and Oxidative Stress

**Figure 4:**
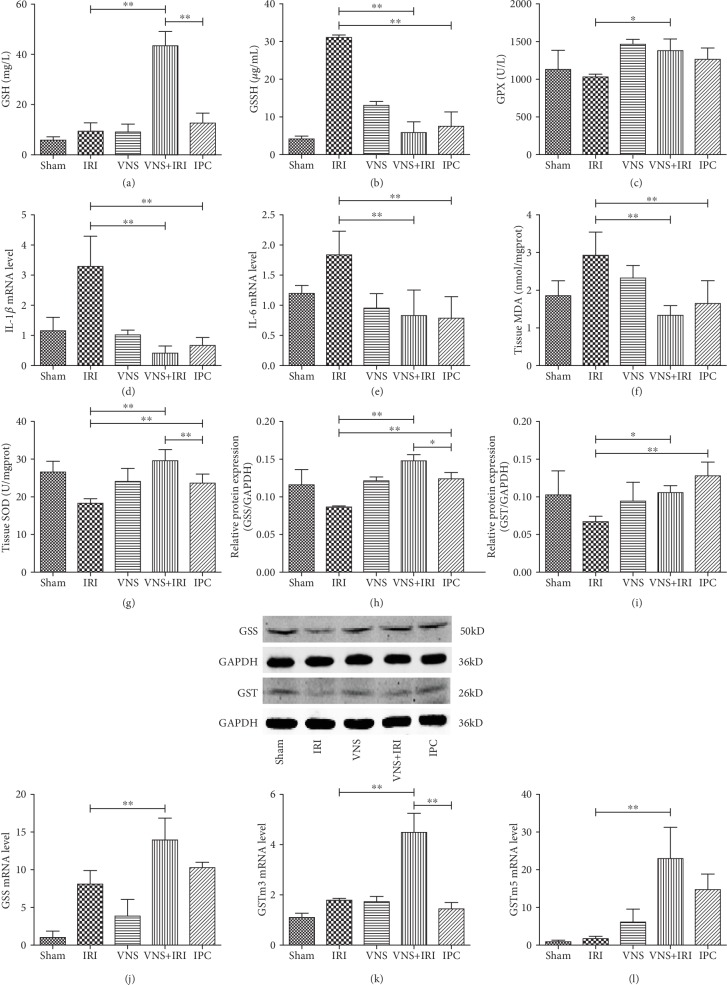
VNS regulated the levels of glutathione and upregulated protein and mRNA levels of glutathione synthetase and glutathione S-transferase. (a) Reduced glutathione, (b) oxidized glutathione, and (c) glutathione catalase activities were detected at the end of 24 h of reperfusion. (d, e) The mRNA expression of IL-1*β* and IL-6 was tested by RT-PCR. (f) Hepatic tissue release of MDA level. (g) Hepatic tissue release of SOD level. (h, i) Western blotting and quantitative analysis show the expressions of glutathione synthetase and glutathione S-transferase proteins. (j–l) RT-qPCR shows the mRNA levels of glutathione synthetase and m3 and m5 glutathione S-transferase. *N* = 5 in each group. ^∗^*P* < 0.05; ^∗∗^*P* < 0.01.

To confirm our analysis of iTRAQ, we detected the levels of GSS, GST, and related metabolites. Hepatic IR induces a considerable reduction in the levels of GSH and GPx activities followed by increases in GSSG levels. However, in Figures 4(a)–4(c), the variations of GSS, GST, and related metabolites of rats in the VNS group, which only received pure electrical stimulation and no hepatic ischemia, were very small, and there were no differences with those in the sham group, suggesting that the VNS alone had no effect on GSS, GST, and related metabolites in normal rats. Since we found that GSS was changed significantly according to iTRAQ and proteomic analysis, we tested whether the protective effect of VNS stimulation during the ischemic period involved variations in glutathione. We found that in the VNS+IRI group, the level of GSH was increased by fourfold compared with the IRI group (Figure 4(a)). Additionally, rats of the VNS+IRI group also had a higher level of GSH compared with the IPC group, which also had close to a 4-fold difference. Glutathione exists in another form in the cytoplasm, as GSSG, so we also evaluated GSSG levels. Consistent with the results of GSH, the VNS+IRI group showed decreases in GSSG compared with the IRI group (Figure 4(b)); GSSG levels of the VNS+IRI group were significantly reduced by almost 6-fold compared with those in the IRI group and also were lower than those in the IPC group, although there was not a statistically significant difference. In addition, livers have complicated antioxidant mechanisms to protect against oxidative stress and maintain redox equilibrium. Among the various antioxidant enzymes, GPx can cooperate with superoxide dismutase (SOD) and catalase to convert oxidized radicals into water. The level of GPx activity was also evaluated. We found that, after stimulation, GPx activity of the VNS+IRI group was enhanced compared with that of the IRI group and was higher than that of the IPC group (Figure 4(c)).

The severity of hepatic IRI is closely related to inflammation and oxidative stress. We found that, among rats in the present model, the livers in the IRI group show a severe increase of inflammation cytokines after reperfusion compared with the sham group. In addition, ischemic liver treatment with VNS significantly reduced IL-1*β* and IL-6 by comparison with the IRI group (Figures 4(d) and 4(e)). Furthermore, as shown in Figures 4(f) and 4(g), IRI livers intervened with VNS considerably increased SOD and reduced MDA levels in comparison with the IRI group.

### 3.6. VNS Improved Glutathione Synthetase and Glutathione S-Transferase Activities in the Liver

Hepatic IRI significantly affected GSH, GSSG, and GPx activity levels (Figure 4), further indicating that the protective effect of VNS was related to glutathione metabolism. Combining previous results, especially proteomic analysis, we examined mRNA and protein expressions of the GSS that was responsible for glutathione biosynthesis to explore why there were lower levels of GSH in livers that underwent IR. We found that the level of GSS protein was prominently increased in the VNS+IRI group compared with the IRI group and was also significantly higher than that in the IPC group. Additionally, the mRNA level of GSS was also augmented remarkably, although there was no discrepancy between the VNS+IRI and IPC groups (Figures 4(h) and 4(j)).

The increased glutathione requires GST to function, and the iTRAQ results hinted that there were two subtypes of GST that had differential expression after VNS. Subsequently, we studied the changing GST subtypes in the VNS-IRI model. Because there were two subtypes of GST, m3 and m5, we examined the mRNA levels of the two genes and the expression of GST protein. We found that, compared with the IRI group, there were remarkable increases in both VNS+IRI and IPC groups, although there was not a statistical discrepancy of protein expression between these two groups (Figure 4(i)). In the VNS+IRI group, vagal stimulation significantly activated GSTm3 compared with both the IRI and IPC groups (Figure 4(k)), as evidenced by the considerably higher GSTm3 mRNA level. Similarly, vagal stimulation enhanced the mRNA level of GSTm5 in the VNS+IRI group after intervention (Figure 4(l)), and it was significantly higher than that in the IRI group. Our study revealed that VNS play a protective role in IRI by regulating protein and mRNA expressions of GSS, GSTm3, and GSTm5.

## 4. Discussion

IR is an inevitable complication of liver surgeries, including LT and resection. Allograft complications, which are influenced by IR, will be early graft failure, high risk of organ rejection, and liver post-reperfusion syndrome (LPRS). Unfortunately, there are still no approved pharmacological treatments for IRI. Thus, effective interventions for IR-induced injury are urgently needed. In the current work, we confirmed that the use of VNS during the ischemic period can prevent IR-induced liver injury. In various trials assessing the protective effects of VNS, a series of parameters, including stimulus intensity, pulse width, frequency, and the duration of stimulation, have been adjusted based on the stimulation effect and specific disease model [40, 41]. In this study, we tried different types of stimulation intensity to optimize the potential therapeutic effect of VNS. In the present work, we found that VNS intervention exerted protective effect against IR-induced impairments. Most importantly, iTRAQ hinted that this result was associated with glutathione metabolism, and our study confirmed that VNS could enhance the production of glutathione and alleviated hepatic tissue damage and hepatocellular apoptosis rate. Mechanistically, the protection of VNS via the upregulation of GSS and GST expressions may have contribution to these profitable effects. Therefore, this study clearly demonstrates that VNS is a protective treatment in hepatic IRI. In addition, the inhibitors of apoptosis proteins (IAPs) are a conserved family of proteins, displaying antiapoptotic properties. These proteins play a direct regulation on the activity of caspases, meaning that IAPs play an important role in cell apoptosis, in oxidative stress, and in immune response. Hence, IAPs may involve in protective function of VNS and the inhibition of apoptosis when glutathione exerts antioxidant function. VNS has also been applied to IRI of the kidney [42], and the results of our proteomic analysis suggested that this regulatory approach is related to glutathione metabolism. We assumed that VNS could serve as a pretreatment before reperfusion, which might reduce inflammation and injury by adjusting the antioxidant capacity of the liver itself.

Moreover, the present research demonstrated that VNS not only reduced hepatic aminotransferase but also ameliorated microstructural damage and apoptosis of hepatic cells. In addition, the protective efficacy of VNS was compared with a common useful intervention, ischemia preconditioning (IPC). Comprehensive analyses indicated that VNS may have had a better therapeutic effect than IPC, signifying that stimulation of the vagus nerve could be a new and effective treatment for IRI. Furthermore, in clinical treatment, IPC is a complicated operation and it needs to be determined when ischemia occurs. Therefore, in contrast to VNS, which can be performed during the ischemia period, IPC is more difficult to implement, and its stringent requirements limit its application. In contrast with IPC, VNS just requires physicians to isolate and stimulate the vagus nerve at the appropriate parameters. In addition, physicians do not need to fully isolate the nerve, especially given recent developments in electrical stimulation instruments [43]. This difference in ease of operation makes VNS more valuable in clinical practice.

**Figure 5:**
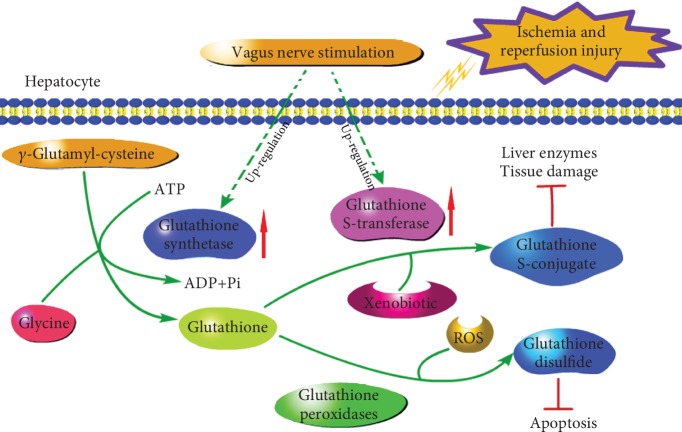
Scheme illustrating the mechanism by which vagus nerve stimulation alleviates hepatic IRI by regulating glutathione production and transformation. Vagal stimulation promotes glutathione biosynthesis in the liver through upregulated glutathione synthetase and a simultaneous increase in the expression of glutathione S-transferase which is a key enzyme responsible for glutathione metabolism.

Judging by hepatic function and histology and the apoptosis rate of hepatocytes at the end of 24 h of reperfusion between the IRI and VNS+IRI groups, a protective effect by VNS was convincing. While the protective effect of VNS was clear, the role and mechanism of VNS in preventing liver IRI remained unclear. To explain our results, we performed proteomic detection, and intriguingly, the expressions of both proteins and mRNA were altered in the VNS+IRI group compared with the IRI group. We found that GSS, GSTm3, and GSTm5 were the main proteins that increased in the VNS+IRI group. The glutathione can maintain the normal metabolism of cells and protect the integrity of the cell membrane, can also inhibit the formation of a fatty liver, and can be combined with the electrophile and free radical and other harmful substances, thereby acting as detoxification and protection of cells from insults. Therefore, the glutathione is one of the key modulators of cellular metabolism. Glutamate-cysteine ligase (GCL) and GSS enzymes are required for glutathione synthesis, and GST is rarely studied. In addition, GST is an enzyme required when glutathione binds to harmful substances such as electrophiles to detoxify and protect cells from insults. Combined with the above results of iTRAQ, both GSS and GST are related to glutathione, which is a common antioxidant. Therefore, we hypothesized that vagus nerve stimulation induces a protective effect and suppresses inflammatory response by upregulating the expression of GSS and GST, which then recover the balance of oxidation and protect the liver. It is known that oxidative stress and its associated inflammation are one of the main pathogeneses of IRI, and the IL-1*β* and IL-6 are two common used marks to evaluate inflammation. We found that IL-1*β* and IL-6 were considerably lower after rats received VNS in the present model. Combined with variations of GSH, GSSG, and activity of GPx, the VNS could protect livers by inhibiting proinflammation cytokines from releasing. In addition, lipid peroxidation also plays a key role in progression of IRI. In the present study, the VNS+IRI group was found having increased SOD and lowered MDA, two common indicators of lipid peroxidation, compared with the IRI group. Results show that the VNS could protect livers from IRI by reducing the degree of lipid peroxidation. Therefore, the VNS may disturb the pathogenesis of IRI and protect livers by oxidation resistance and detoxification of GSH. Indeed, hepatic IRI has been shown to reduce GSH levels [44], and GSH plays an important role in the pathogenesis and progression of IRI [45]. Compared with the IRI group, the level of GSH of VNS+IRI was significantly increased after being stimulated and was higher than that in the IPC group, meaning that VNS could promote more reduced glutathione generation than IPC to scavenge free radicals. Furthermore, we also found that plasma levels of GSSG and GPx activity were significantly reduced and increased, respectively, after vagal stimulation. From the above results, there is significant positive correlation between levels of GSH, GPx activity, and VNS; similarly, there is an inverse correlation between levels of GSSG and VNS. Consistent with these results, several studies have documented that there is a link between oxidative stress and diverse liver diseases [46, 47]. Therefore, the VNS reversal of the observed liver injuries induced by IRI in our study further supports the hypothesis that hepatic protection by VNS operates mainly via activation of glutathione metabolism. In this pathway, VNS protects hepatic tissues from IRI by activating the GSS-GSH-GST reflex (Figure 5).

In this glutathione metabolism pathway, GSS is a key component that protects against IR injury [48]. Another critical molecule of glutathione metabolism is GST, which mediates glutathione for detoxification [49]. In this signaling process, GSS probably plays a more important role than GST in modifying glutathione metabolism. GSS is now regarded as a key synthetase that regulates the plasma level of GSH and catalyzes the reaction that ultimately forms glutathione [50]. It promotes the biosynthesis of glutathione and catalyzes the reaction of *γ*-glutamyl-cysteine and glycine to form glutathione. Our findings show that VNS can protect livers from IRI by upregulating the mRNA and protein expressions of GSS and GST after VNS. These results reveal the basic mechanism by which the vagus nerve mediates pivotal and therapeutic effects in alleviating hepatic IR-induced injuries.

Although a moderate inflammatory response is beneficial to the body, persistent or excessive inflammation can lead to injuries and diseases, such as IRI. Therefore, reducing inflammation is crucial. Recent studies have found that CAP is a new inflammatory regulatory mechanism [16, 17]. CAP can conduct an anti-inflammatory signal and travel through the efferent fibers of the vagus nerve; these signals reach the organs which have the reticular endothelial system, such as the spleen, liver, and heart, and then release acetylcholine (Ach), binding with macrophages and other immune cells on the expression of alpha 7 nicotine-type acetylcholine receptor (*α*7nAChR) to inhibit proinflammatory cytokines from releasing, thus acting as anti-inflammatory and analgesic agents [18]. It is proved that PUN-282987, which is specific agonist for *α*7nAChR, can upregulate the phosphorylation level of the extracellular signal-regulating kinase (ERK) and that methyllycaconitine, which is antagonist for *α*7nAChR, can inhibit the phosphorylation of ERK. In addition, activation of *α*7nAChR can inhibit lipopolysaccharide- (LPS-) induced phosphorylation of P38 [51]. These results suggest that the mitogen-activated protein kinase (MAPK) pathway may be involved in the CAP pathway. Therefore, the MAPK (for instance, p38, ERK) signaling pathway could represent a potential regulation pathway by VNS, and the spleen may also involve in these.

Alleviating IRI has significant clinical implications; reducing IRI can conspicuously reduce the incidence of postoperative complications and a patient's financial burden. Hence, this goal has attracted increasing attention. In recent decades, there have been numbers of advancements in treatment of IRI. However, there is no method to alleviate IRI without tedious or invasive operations. This work reveals that VNS is a well-tolerated method to significantly decrease inflammation, reperfusion-induced tissue damage, disordered plasma levels of reduced and oxidized glutathione, and apoptosis that are caused by IR. VNS may become a novel, lower-trauma, and even noninvasive therapy for treating patients suffering from IRI, although large randomized clinical trials are first needed.

It is important to mention that there were still several limitations in our work. In exploring the underlying mechanism of VNS alleviating the liver IRI, we did not intervene the protein and mRNA expressions of GSS and GST. Besides, although the rat ischemic model is recognized as the ideal method, however, compared with the animal model, *in vitro* experiments still have some advantages, which can reduce variation within groups. Therefore, we will further research on the glutathione metabolism and the protective effect of VNS. Meanwhile, VNS applied to the vagus nerve is a systemic intervention method, and previous studies also have confirmed that the spleen can participate in the CAP pathway activated by VNS to alleviate IRI. Therefore, whether VNS promotes glutathione synthesis in the liver related to the spleen and whether VNS alleviates IRI related to inflammatory cells will be investigated in the near future.

## 5. Conclusions

We demonstrated that short-term VNS could exert a protective effect against hepatic dysfunction in a rat model with 70% hepatic IR. The underlying mechanism by which VNS conferred its protective effects during ischemia was in part by activation of antioxidant GSH by the GSS-GSH-GST reflex. Through this pathway, VNS contributed to the attenuation of the liver damage and apoptosis caused by IRI in ischemic rat livers.

## Data Availability

The data used to support the findings of this study are available from the corresponding authors upon request.

## Supplementary Materials

Supplementary Materials Figure S1: the complete western blots of glutathione synthetase and glutathione S-transferase proteins. (A) Glutathione synthetase. (B) Glutathione S-transferase.
