# Supplementary material for: Vagus Nerve Stimulation Alleviates Hepatic Ischemia and Reperfusion Injury by Regulating Glutathione Production and Transformation
Source: Oxid Med Cell Longev. 2020 Jan 21;2020:1079129. doi: 10.1155/2020/1079129 (PMC6996675; doi:10.1155/2020/1079129)
Supplement: Supplementary Materials — Figure S1: the complete western blots of glutathione synthetase and glutathione S-transferase proteins. (A) Glutathione synthetase. (B) Glutathione S-transferase. [file 1079129.f1.pdf]

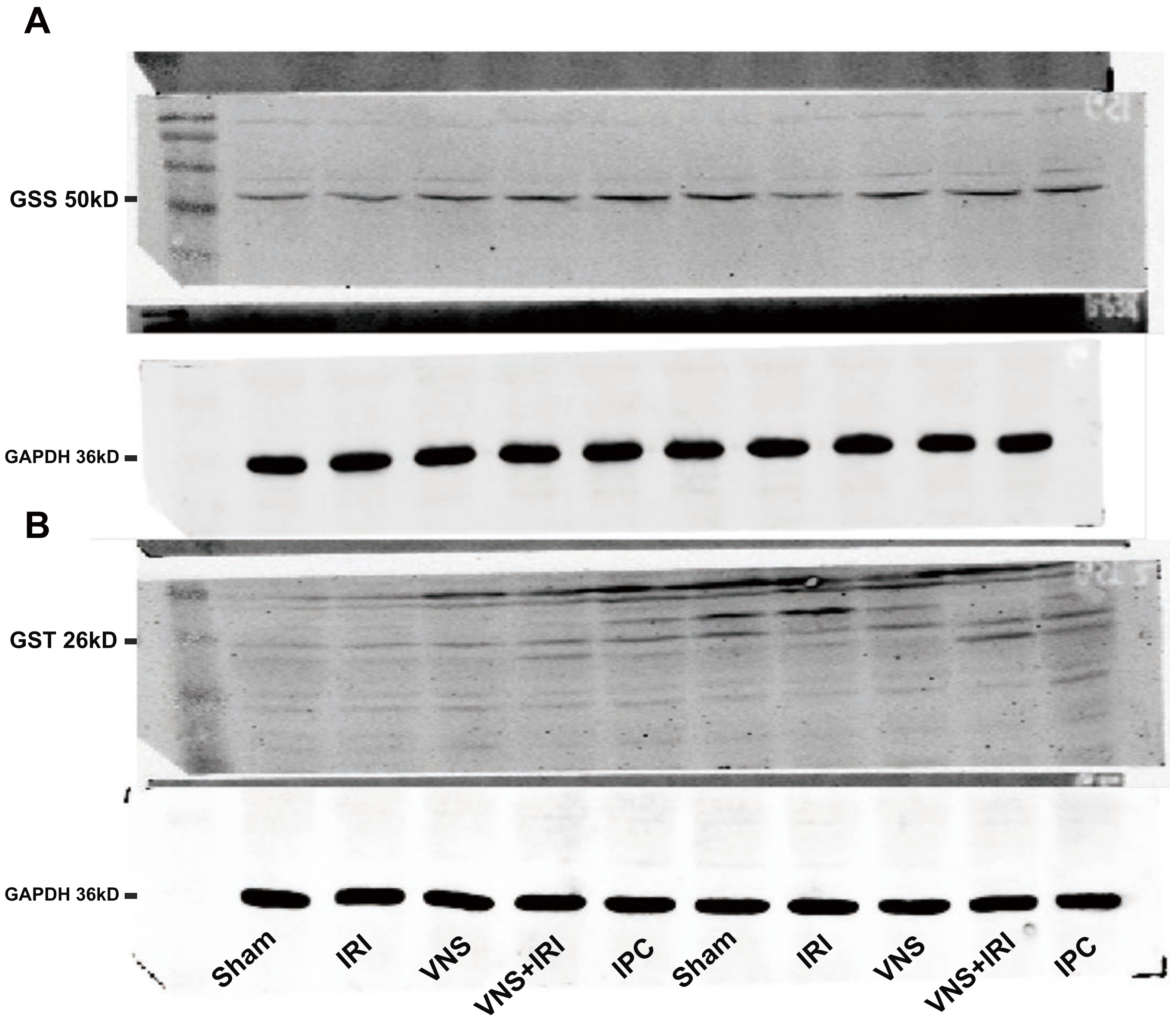
A
GSS 50kD
GAPDH 36kD
B
GST 26kD
GAPDH 36kD
Sham
IRI
VNS
VNS+IRI
IPC
Sham
IRI
VNS
VNS+IRI
IPC

Figure S1. The complete western blots of glutathione synthetase and glutathione S-transferase proteins. (A) Glutathione synthetase. (B) Glutathione S-transferase.
